# Convolutional neural network based on automatic segmentation of peritumoral shear-wave elastography images for predicting breast cancer

**DOI:** 10.3389/fonc.2023.1099650

**Published:** 2023-02-14

**Authors:** Li Xie, Zhen Liu, Chong Pei, Xiao Liu, Ya-yun Cui, Nian-an He, Lei Hu

**Affiliations:** ^1^ Department of Ultrasound, The First Affiliated Hospital of University of Science and Technology of China (USTC), Division of Life Sciences and Medicine, University of Science and Technology of China, Hefei, Anhui, China; ^2^ Department of Computing, Hebin Intelligent Robots Co., LTD., Hefei, China; ^3^ Department of Respiratory and Critical Care Medicine, The First People’s Hospital of Hefei City, The Third Affiliated Hospital of Anhui Medical University, Hefei, China

**Keywords:** convolutional neural networks (CNN), shear-wave elastography (SWE), peritumoral stiffness, segmentation, breast cancer

## Abstract

**Objective:**

Our aim was to develop dual-modal CNN models based on combining conventional ultrasound (US) images and shear-wave elastography (SWE) of peritumoral region to improve prediction of breast cancer.

**Method:**

We retrospectively collected US images and SWE data of 1271 ACR- BIRADS 4 breast lesions from 1116 female patients (mean age ± standard deviation, 45.40 ± 9.65 years). The lesions were divided into three subgroups based on the maximum diameter (MD): ≤15 mm; >15 mm and ≤25 mm; >25 mm. We recorded lesion stiffness (SWV1) and 5-point average stiffness of the peritumoral tissue (SWV5). The CNN models were built based on the segmentation of different widths of peritumoral tissue (0.5 mm, 1.0 mm, 1.5 mm, 2.0 mm) and internal SWE image of the lesions. All single-parameter CNN models, dual-modal CNN models, and quantitative SWE parameters in the training cohort (971 lesions) and the validation cohort (300 lesions) were assessed by receiver operating characteristic (ROC) curve.

**Results:**

The US + 1.0 mm SWE model achieved the highest area under the ROC curve (AUC) in the subgroup of lesions with MD ≤15 mm in both the training (0.94) and the validation cohorts (0.91). In the subgroups with MD between15 and 25 mm and above 25 mm, the US + 2.0 mm SWE model achieved the highest AUCs in both the training cohort (0.96 and 0.95, respectively) and the validation cohort (0.93 and 0.91, respectively).

**Conclusion:**

The dual-modal CNN models based on the combination of US and peritumoral region SWE images allow accurate prediction of breast cancer.

## Introduction

The morbidity of breast cancer in Asian women with dense breasts is even 4–6 times higher than that in western women with fatty breasts (1–3). Breast ultrasound (US) has been recognized as the main imaging method for diagnosing breast cancer (4–6). American College of Radiology Breast Imaging Reporting and Data System(ACR-BIRADS) is used for the evaluation and follow-up recommendations of breast lesions detected by US. However, radiologists’ subjective classification differences may affect diagnostic performance, thereby leading to overtreatment, especially for lesions with BI-RADS 4 category (5, 6).

Although shear-wave elastography (SWE) may improve the diagnostic specificity of the conventional US for breast cancer, even in cases of small or interval breast cancer (IBC) (4–7). However, due to the inhomogeneity within the lesion (hemorrhage, calcification, and cystic appearance) introduces subjective bias, which influences the final measured value, the specificity remains limited up to 86% when the quantitative SWE parameters were used, thereby may lead to unnecessary biopsies (5–8)

Previous studies have confirmed that the assessment of peritumoral stiffness of breast lesions can improve the accuracy of SWE in predicting breast cancer, considering desmoplastic reaction and tumor cell infiltration into the peritumoral stroma (9–12). Moreover, the peritumoral invasion is an independent prognostic factor significantly associated with an increased risk of relapse and death in node-negative breast cancer patients (13, 14). However, it is difficult to distinguish the boundary between the normal and tumor tissue in SWE (13). Therefore, peritumoral stiffness of breast lesions is highly dependent on radiologists’ experience rather than on the integrated high-throughput imaging information (12–14). Thus, it is desirable to develop approaches using artificial intelligence (AI) to integrate high-throughput imaging information that cannot be directly identified by unaided eye, so as to offer assistance to radiologists and improve the efficiency and accuracy of breast cancer diagnosis.

Recently, deep convolutional neural network (CNN)-based approaches have been considered as an effective approach for the feature extraction and classification of US images in breast cancer diagnosis (15–18). However, most of the CNN models used in the diagnosis of breast cancer have been based on the US or SWE images of intratumoral tissue rather than peritumoral tissue (15–20). The peritumoral stiffness of breast lesions is an accurate predictor of breast cancer (9–12). However, based on the traditional SWE technology, it is difficult to obtain accurate SWE image of the peritumoral tissue, and it is hard to estimate which width of the peritumoral tissue should be evaluated to provide the optimal diagnostic index of benign and malignant lesions (13).

Few studies have used CNN-based AI diagnostic systems to predict breast cancer based on peritumoral region’s SWE image. Therefore, the purpose of this study was to develop a dual-modal CNN model based on peritumoral SWE image of breast lesions and examine its diagnostic performance in breast cancer. The dual-modal CNN model was able to automatically recognize the location of breast lesions in B-mode US images. After mapping the lesions’ boundaries detected on B-mode US images to SWE images, segmentation of different widths (0.5 mm, 1.0 mm, 1.5 mm, 2.0 mm) of the peritumoral tissue was automatically completed. Then, we evaluated the predictive performance of each CNN model based on different peritumoral widths for breast cancer.

## Materials and methods

### Study population

**Figure 1:**
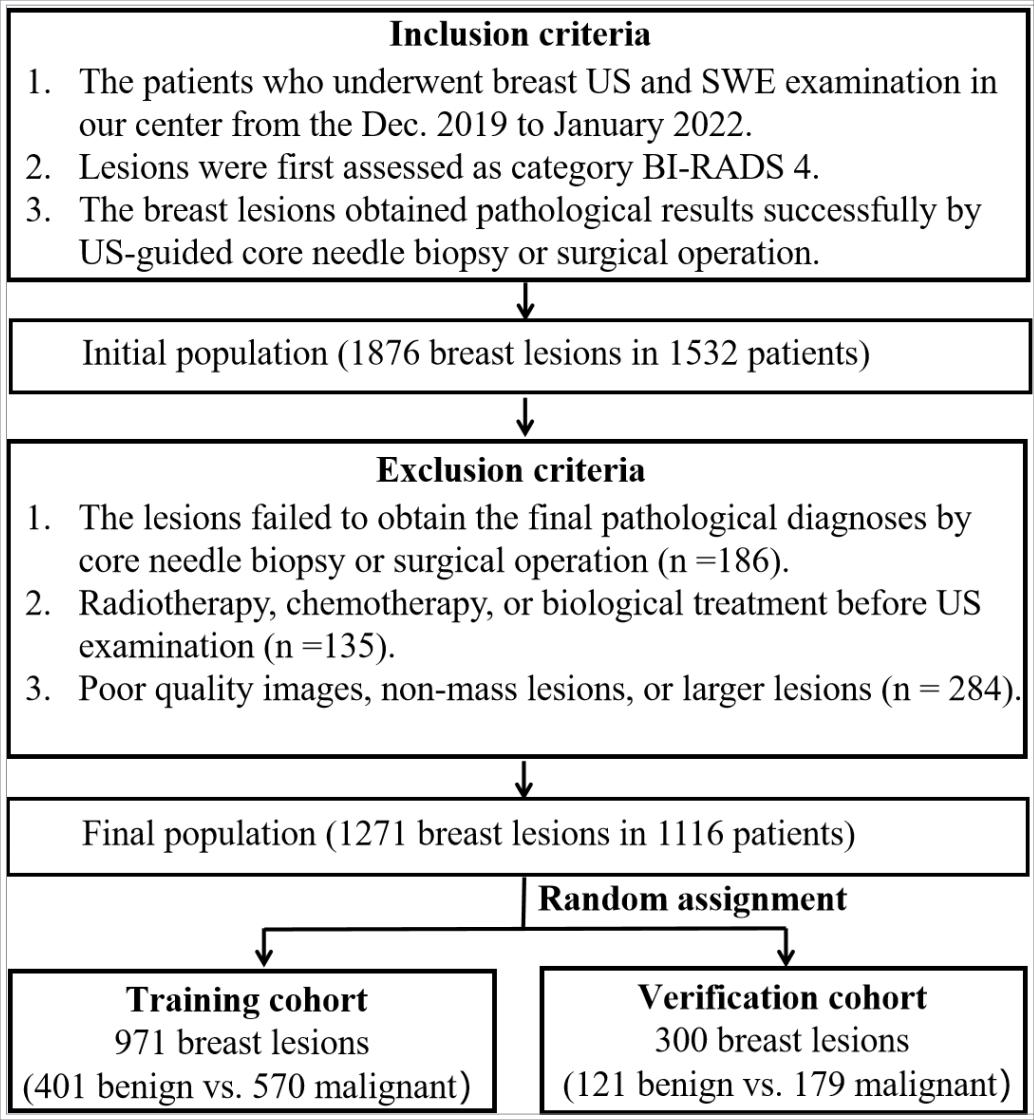
Flowchart of breast lesions recruitment.

This retrospective study was approved by the institutional ethics committee of the First Affiliated Hospital of the University of Science and Technology of China (USTC). Between December 2019 and April 2022, the initial population included 1876 breast lesions in 1532 consecutive patients who had undergone US and SWE examinations. The inclusion criteria were as follows: (i) ACR-BIRADS 4 category of breast lesions; (ii) solid or cystic solid breast lesions examined by B-mode US and SWE; (iii) core needle biopsy or surgical resection performed to obtain accurate pathological results. The exclusion criteria were as follows: (i) radiotherapy, chemotherapy, or biological treatment before US examination; (ii) a history of breast surgery (including excision or plastic surgery); (iii) pregnancy or lactation; (iiii) non-mass lesions or larger lesions (larger than 40 mm), which were beyond the maximum range of the SWE sampling frame. Finally, a total of 1271 ACR-BIRADS 4 lesions from 1116 female patients were analyzed in this study. The lesions were assigned to the training cohort (971 lesions) and the verification cohort (300 lesions) by random sampling at an approximate ratio of 3:1 (**Figure 1**).

### Dual-modal image acquisition and preprocessing

All of the US and SWE examinations were performed by breast radiologists using the Siemens ACUSON Sequoia (Siemens Healthcare GmbH, USA) US system equipped with 10 MHz linear array transducers. We acquired and stored transverse and longitudinal static images with the maximum diameter of breast lesions on US, and images containing the lesion’s characteristics (such as calcification, angulation, and spiculation sign) were also stored. All breast lesions included in this study were of ACR-BIRADS 4 category. The category of all lesion was reassessed by two radiologists both with more than eight years of working experience, and another radiologist with 12 years of breast examination experience was consulted to reach a final decision when disagreements occurred.

**Figure 2:**
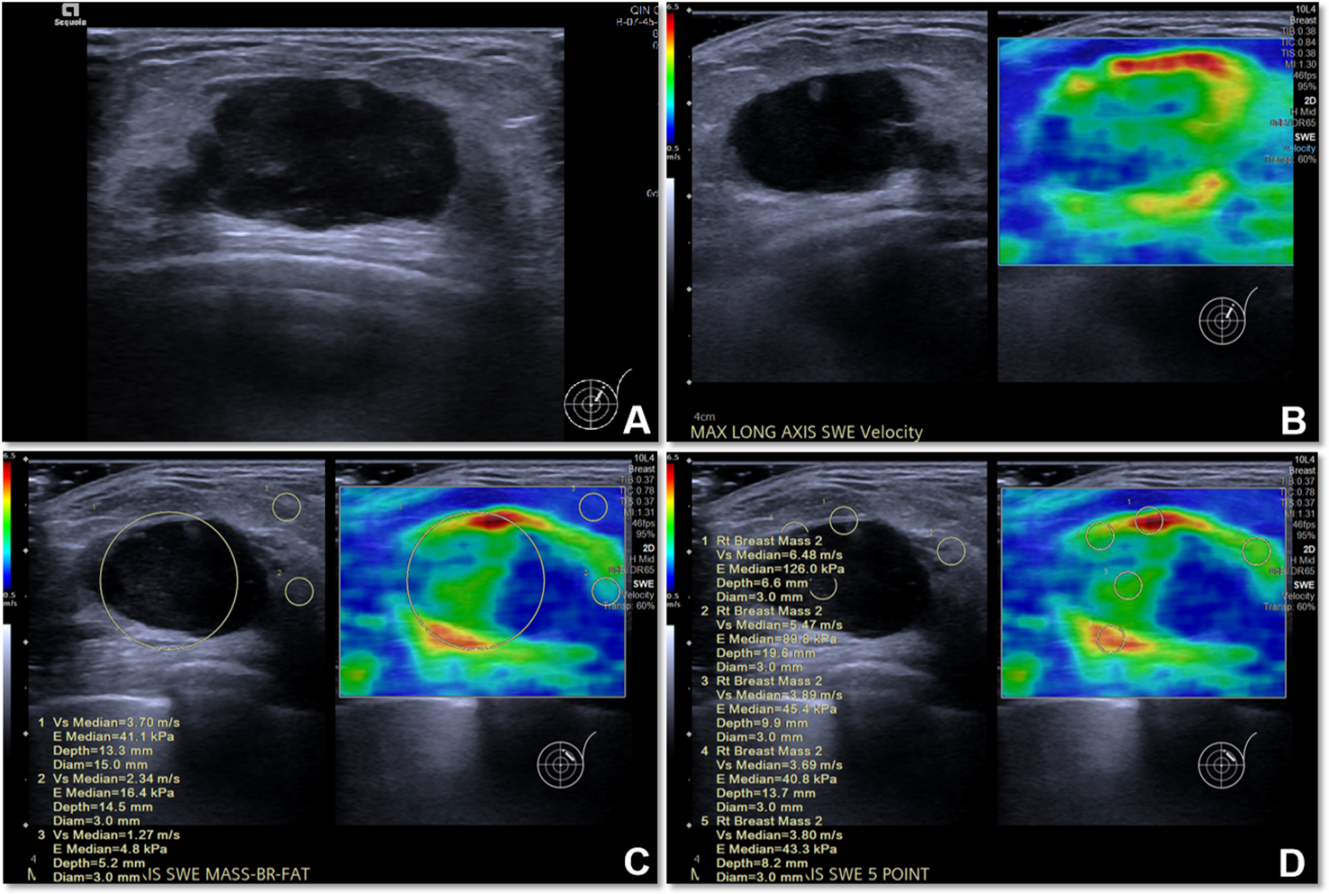
The US image and quantitative SWE parameters of metaplastic carcinoma (sarcomatoid carcinoma) in a 77-year-old woman. **(A)** US image of the lesion. **(B)** SWE image of the lesion. **(C)** The SWV1 value of the lesion was 3.70 m/s, while that of the normal mammary gland was 2.34 m/s and that of adipose tissue was 1.27 m/s. **(D)** The SWV5 value of the lesion and peritumoral region was 4.66 m/s.

SWE examination of each lesion was performed in machine default modes, and we adjusted the size of a rectangular region of interest (ROI) to cover the whole lesion. We stored a static SWE image of the lesion and the surrounding tissue without measurement (for image segmentation); the lesion was put in the middle of the SWE region ensuring that the ROI included the lesion and at least 5 mm of the surrounding breast tissue. Then, quantitative SWE parameters were measured as follows: (i) a round ROI containing the lesion was measured and its shear-wave velocity (SWV) was recorded as SWV1, which represents the average stiffness of the lesion; (ii) five 3-mm-wide round ROIs were selected for measurement; four of them were placed at locations adjacent to the lesion (including peritumoral and intratumoral tissues), and one ROI was placed inside of the lesion, and the average SWV was recorded as SWV5, which represents the average stiffness of the lesion including the peritumoral tissue (**Figure 2**). All SWE images were taken from the longitudinal and transverse section of the breast lesions, and two senior breast radiologists with more than eight years of clinical experience performed all of the SWE examinations. The radiologists in this study were blinded to the patients’ clinical data and pathological results. All of the US and SWE images extracted from the Siemens database sites were in.jpg format. In order to determine whether the size of lesions has an impact on the selection of optimal peritumoral region SWE images of the lesions, in this study we divided the lesions to three subgroups depending on their maximum diameter (MD) (≤15 mm; >15 mm and ≤25 mm; >25 mm).

All of the breast lesions were pathologically confirmed through US-guided core needle biopsy or surgical pathology after US and SWE examination. Benign lesions were followed-up with US for at least 18 months and showed no increase in the maximum diameter and volume on conventional US.

### Labeling and segmentation of the peritumoral region

We constructed a CNN segmentation model for segmentation of lesions on SWE images. Traditionally, color SWE images are often not suitable for deep learning or induce instability because boundaries of lesions are difficult to label. Therefore, for the segmentation of the interior of the lesion, we labeled the SWE images according to the lesion boundary on the US image. First, we pretrained two backbone models of PP-LiteSeg and EfficientNet-B0 using Simsiam network architecture to identify the lesions on US images. Simsiam is a siamese network that has shown to be an effective self-supervised method. We used 83590 non-annotated US images of breast lesions for pretraining. Then, the lesion’s region in each US image in the training cohort was manually labeled using an open-source annotation tool (Labelme, https://github.com/wkentaro/labelme) by one radiologist (with eight years of experience in breast US) who was blinded to the clinical data and histopathological results of the patients.

After training, we obtained a segmentation model for describing the shape of the lesion on US images. The segmentation model allowed us to draw an ROI encompassing breast lesion’s boundary on the two-dimensional image, and then map the lesion’s boundary onto the SWE image and automatically expand the boundary of the peritumoral tissue with different widths (0.5 mm, 1.0 mm, 1.5 mm, 2.0 mm). For each SWE image of breast lesions, the following six images were finally segmented: the interior of the lesion; the lesion including 0.5 mm of peritumoral tissue; the lesion including 1.0 mm of peritumoral tissue; the lesion including 1.5 mm of peritumoral tissue; the lesion including 2.0 mm of peritumoral tissue; and the whole rectangular SWE ROI image.

**Figure 3:**
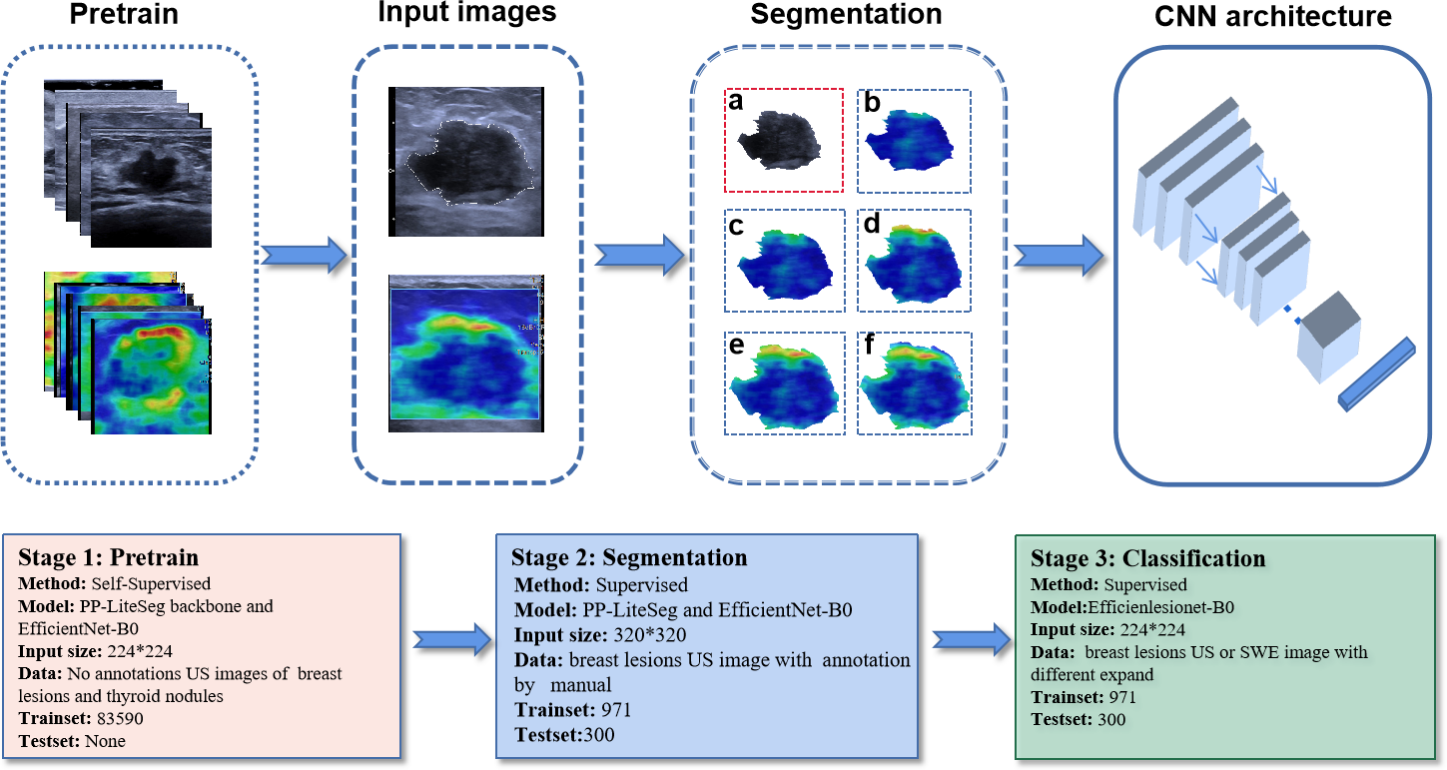
The process of model pretraining and segmentation network construction. The trained segmentation model automatically segments the original US and SWE images into six standard data inputs to classification models: **(A)** the segmented US image of the lesion; **(B)** the segmented SWE image of the lesion; **(C)** the segmented SWE image of the lesion including 0.5 mm of peritumoral tissue; **(D)** the segmented SWE image of the lesion including 1.0 mm of peritumoral tissue; **(E)** the segmented SWE image of the lesion including 1.5 mm of peritumoral tissue; **(F)** the segmented SWE image of the lesion including 2.0 mm of peritumoral tissue.

The segmentation model comprised encoder, aggregation, and decoder. It was implemented on PaddlePaddle (https://github.com/PaddlePaddle/PaddleSeg). Compared with the default config, we removed some data augmentations including ResizeStepScaling and RandomPaddingCrop, added rotation augmentations, modified the num_classes to 2, and limited input_size to 320×320. OHEM loss was selected according to the better performance in a small target binary segmentation than cross-entropy loss. The process of model pretraining and segmentation network construction is shown in **Figure 3**.

To achieve high consistency of the automatic segmentation model, the Dice similarity coefficient, Cohen’s kappa, Hausdorff 95 (95% HD), and the segmentation metrics (area, major axis length, and minor axis length) were used to evaluate each lesion’s pixel and boundary consistency of the three radiologists and the CNN segmentation model in the validation cohort. Each of the three radiologists had eight years of experience in breast US and was blinded to the clinical data and histopathological results of the patients.

### CNN-based predictive model building based on the single-segmentation peritumoral region SWE image

**Figure 4:**
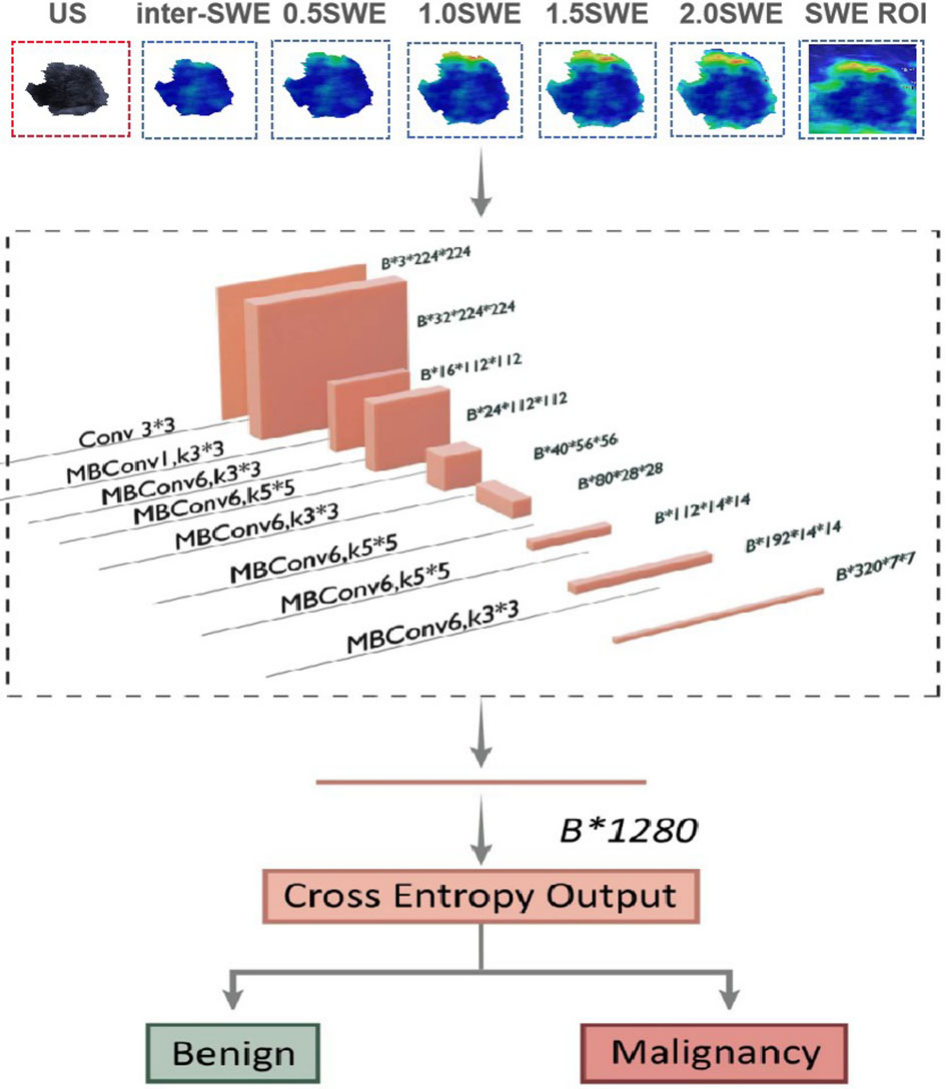
Network architecture of single prediction model for breast cancer. The input layer is a single US image or a segmented SWE image.

For predicting breast cancer, we built benign and malignant binary classification CNN models by EfficientNet-B0. Seven single-parameter CNN models were trained based on seven individual input images (**Figure 4**). Five of them were segmented on SWE (Internal SWE CNN, 0.5 mm SWE CNN, 1.0 mm SWE CNN, 1.5 mm SWE CNN, 2.0 mm SWE CNN); one was segmented on US image; and the last one was the whole heat map region on SWE image. EfficientNet-B0 architecture is shown in **Figure 4**. The optimizer was SGD with 0.9 momentum and Le-4 weight-decay; the loss function was cross entropy loss; batch was 128; and the learning rate was 0.01. Data augmentation included random horizontal flip, random brightness, random contrast, and random saturation. We implemented them in an open-source machine learning framework (PyTorch, version is 1.10, https://pytorch.org).

### CNN-based predictive model building based on the fusion of peritumoral region segmentation SWE images and US images

**Figure 5:**
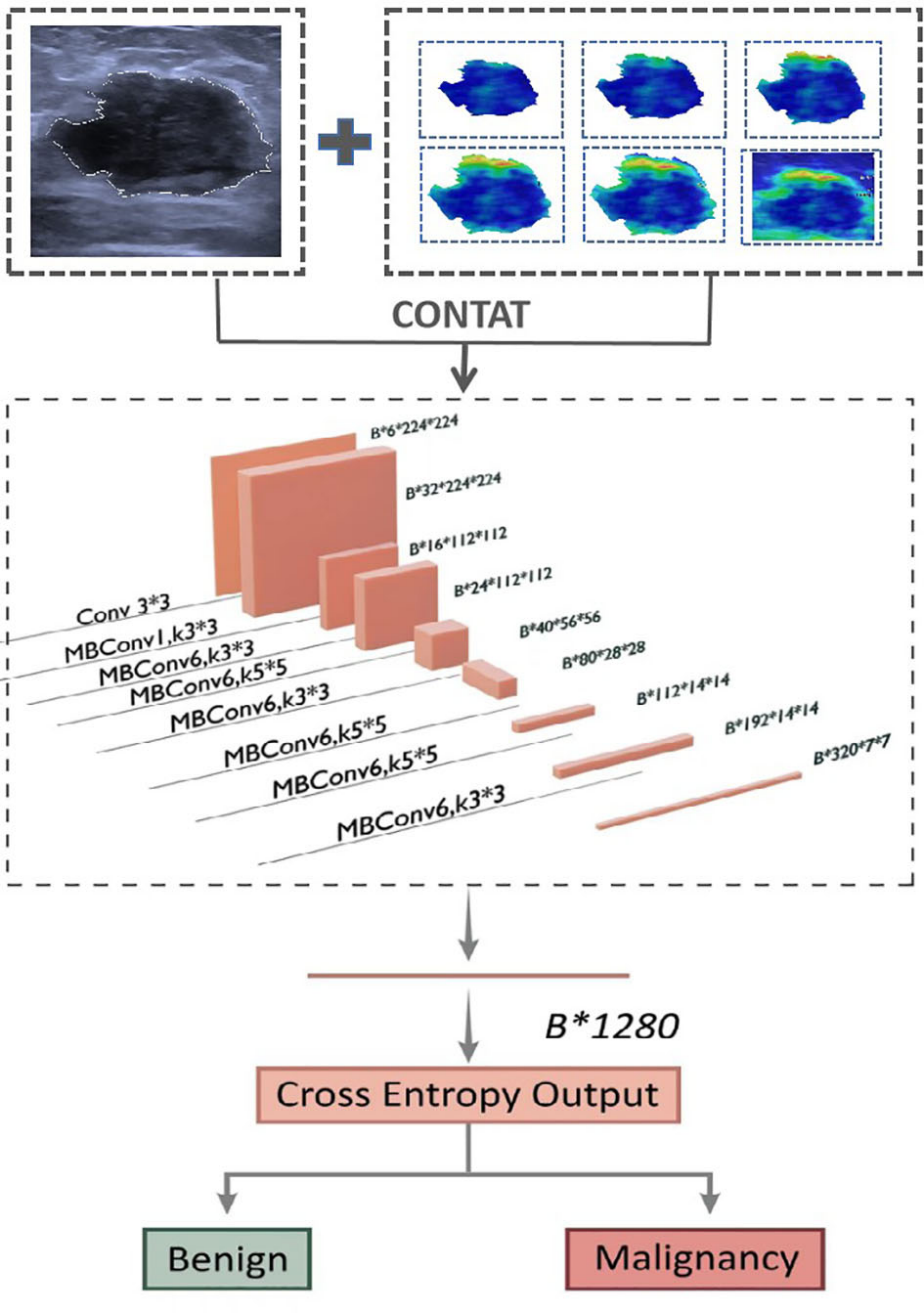
Network architecture of dual-modal prediction model for breast cancer. The input layer inp.

Furthermore, six corresponding US + SWE image CNN models (US + Internal SWE CNN; US + 0.5 mm SWE CNN; US + 1.0 mm SWE CNN; US + 1.5 mm SWE CNN; US + 2.0 mm SWE CNN; US + ROI SWE CNN) were also trained based on the fusion-input binary classification CNN by using both US and SWE images as the network input. We fused the two images on the first convolution layer of the network. For the fusion input, we modified the EfficientNet-B0, which increased input channel of the first convolution layer from 3 to 6. Correspondingly, two three-channel images were concatenated into six channels on the channel axis and then resized into 224×224. Other parameters of the model were the same as before, and the model architecture is shown in **Figure 5**.

### Statistical analysis

Statistical analysis was performed using commercially available SPSS software (version 19.0; Chicago, USA). All numerical data were presented as the mean ± standard deviation. The Shapiro–Wilk test was used to verify whether the quantitative data were normally distributed. The Mann–Whitney *U* tests were used to compare the continuous variables between the benign and malignant groups or between the three subgroups in the training and validation cohorts. To evaluate the consistency between the radiologists and the CNN segmentation model segmentation, we utilized the sensitivity, specificity, Dice coefficient, Cohen’s kappa, and 95% Symmetric Hausdorff Distance. The Pearson correlation coefficients and Wilcoxon signed-rank tests were used to determine whether the CNN segmentation model’s performance aligned with that of the radiologists. We used three metrics (area, length of major axis, and length of minor axis) to evaluate the consistency of segmentation. The performances of all predictive CNN models and two quantitative SWE parameters were assessed using receiver operating characteristic (ROC) curve analysis. ROC was also used to calculate the corresponding sensitivity (SEN), specificity (SPE), positive predictive value (PPV), negative predictive value (NPV), and area under the ROC curve (AUC). The McNemar test was used for paired comparisons of proportions. All statistical tests were two-sided, and p values lower than 0.05 were considered to indicate statistical significance.

## Results

### Clinicopathologic characteristics of all breast lesions

The clinicopathologic characteristics and subgroups of all 1271 breast lesions are summarized in **Table 1**. Among the 1271 BI-RADS 4 lesions (ACR-BIRADS 4A:397; ACR-BIRADS 4B: 575; ACR-BIRADS 4C: 299), 749 (58.9%) were malignant and 522 (41.1%) were benign, as shown in **Table 2**. The mean age of the entire cohort was 45.40 ± 9.65 years (range, 19–79 years); the mean age in the malignant group was greater than that in the benign group (51.29 ± 7.29 vs. 39.56 ± 6.73, *P* < 0.001), and there was no significant difference in the mean age between the training and the validation cohorts (*P* > 0.05 for all). The MD of the malignant group was larger than that of the benign group (19.98 ± 5.22 mm vs. 14.55 ± 7.74 mm, *P* < 0.001), but there was no difference between the training cohorts and the validation cohorts (*P* > 0.05 for all). The lesions were divided into subgroups depending on the MD. The subgroup with the MD of lesions ≤15 mm included 218 lesions (167 in the training cohort and 51 in the validation cohort; 89 benign and 129 malignant); the subgroup with MD between 15 mm and 25 mm included 779 lesions (598 in the training cohort and 181 in the validation cohort; 327 benign and 452 malignant); and the subgroup with MD >25 mm included 274 lesions (206 in the training cohort and 68 in the validation cohort; 112 benign and 162 malignant).

**Table 1:** Distribution of subgroups studied and quantitative SWE parameters of all lesions.

	Total	Benign	Malignant	Training cohort	Validation cohort	Subgroup A MD≤15mm	Subgroup B 15mm≤MD≤25mm	Subgroup C MD>25mm	ACR-BIRADS 4A	ACR-BIRADS 4B	ACR-BIRADS 4C
Age (year)	45.40 ± 9.65	39.56 ± 6.73	51.29 ± 7.29	47.10 ± 6.65	46.87 ± 7.54	47.33 ± 7.45	46.98 ± 6.11	47.28 ± 9.29	44.73 ± 7.55	44.98 ± 6.11	46.28 ± 6.99
Lesions (n)	1271	522	749	971	300	218	779	274	397	575	299
Maximum diameter (mm)	17.45 ± 8.51	14.55 ± 7.74	19.98 ± 5.22	18.55 ± 7.74	17.67 ± 6.42	^_^	^_^	^_^	15.99 ± 7.74	15.98 ± 5.22	19.59 ± 3.34
B *vs.* M	522 *vs*.749	^_^	^_^	401 *vs*. 570	121 *vs*. 179	89 *vs.*129	327 *vs.*452	112 *vs.* 162	350 *vs.* 47	171 *vs.* 404	1 *vs.* 298
T *vs.* V	971 *vs.*300	401 *vs*.121	570 *vs*. 179	^_^	^_^	167 *vs.*51	598 *vs.*181	206 *vs.*68	157 *vs.*51	618 *vs.*189	196 *vs.*60
SWV1 (m/s)	2.67 ±1.87	1.85±0.65	3.76±0.78	2.65±1.34	2.71±1.52	2.48±1.35	2.98±0.98	2.65±1.08	2.04±1.15	2.89±1.98	3.52±2.08
SWV5 (m/s)	2.98 ±1.65	1.67±0.74	4.02±0.82	2.91±1.42	3.05±1.17	2.66±1.12	3.21±1.02	2.73±0.97	1.91±0.79	3.22±1.98	4.03±2.08
*P* value	0.000	0.000	0.000	0.000	0.000	0.000	0.000	0.000	0.000	0.000	0.000

MD, maximum diameter of lesions; T, training cohort; V, validation cohort; B, benign; M= malignant; ^_^ indicates not applicable; ACR-BIRADS: American College of Radiology Breast Imaging Reporting and Data System; P value: Compared with SWV1 and SWV5.

**Table 2:** Summary of pathologic findings.

Histopathologic results		n (%)
Benign (n = 522)	Fibroadenoma	206 (39.5)
	Adenosis	136 (26.1)
	Intraductal papilloma	59 (11.4)
	Adenosis with Fibroadenoma or Intraductal papilloma	54 (10.3)
	Others^#^	67 (12.7)
Malignant (n =749)	Invasive ductal carcinoma	556 (74.3)
	Ductal carcinoma in situ	84 (11.2)
	Invasive lobular carcinoma	54 (7.3)
	Intraductal papillary carcinoma	20 (2.6)
	Mucinous carcinoma	13 (1.7)
	Others*	22 (2.9)

Others#=Interstitial lesions (n=19); Inflammatory lesions (n=17); Fibroepithelial tumor(n=13); Interstitial hyperplasia with hyaline degeneration(n=11); Phyllodes tumor(n=5) Diffuse large B-cell lymphoma (n=2). Others*=Adenoid ductal carcinoma in situ (n=6); Poorly differentiated adenocarcinoma (n=5); Lobular carcinoma in situ (n=5); Mixed ductal and lobular carcinoma (n=3); Mucinous carcinoma with ductal carcinoma in situ (n=2); Metaplastic carcinoma (sarcomatoid carcinoma) (n=1).

### Quantitative SWE parameters of all breast lesions

Considering 1271 lesions, SWV values of the malignant group were significantly higher than those of the benign group, including the intratumoral stiffness (SWV1: 3.76 ± 0.78 m/s vs. 1.85 ± 0.65 m/s; *P* < 0.01) and the peritumoral stiffness (SWV5: 4.02 ± 0.82 m/s vs. 1.67 ± 0.74 m/s; *P* < 0.01).

SWV5 values were significantly higher than SWV1 values in the malignant group, ACR-BIRADS 4B and 4C group; SWV5 values were lower than SWV1 values in the benign group and ACR-BIRADS 4A group (all *P* < 0.01). SWV5 and SWV1 values in the subgroup with 15 mm <MD ≤25 mm were higher than those in the subgroups with MD ≤15 mm and MD >25 mm, because there were more malignant lesions in this subgroup (all *P* < 0.01).

### Consistency analysis of lesion segmentation manually vs. CNN model

**Figure 6:**
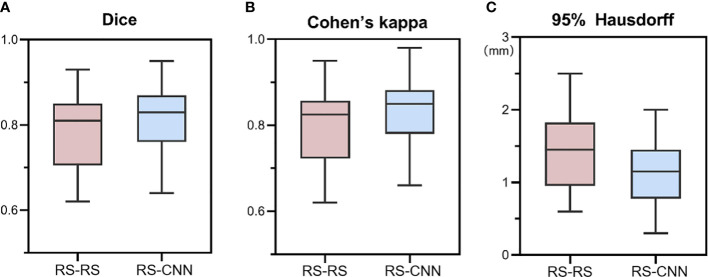
Box-plot diagrams of Dice **(A)**, Cohen’s kappa **(B)**, and 95% Hausdorff metrics **(C)** compared between the radiologists’ segmentation (RS-RS) and between the experts and deep learning model segmentation (RS-CNN). RS, radiologists; CNN, convolutional neural network.

**Figure 7:**
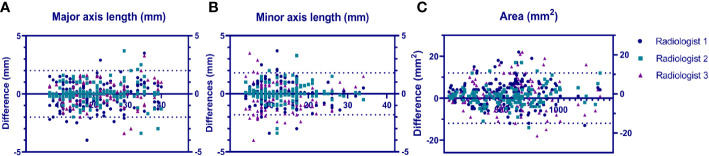
Bland–Altmann plots showing the model performance vs. that of each radiologist in terms of lesion major axis length **(A)**, lesion minor axis length **(B)** and lesion area **(C)**.uts a US image and a segmented SWE image at the same time.

We calculated the average values of the segmentation metrics’ intra- and interrater consistency of the three radiologists, as shown in **Table 3**. Wilcoxon signed-rank tests were conducted, and Pearson’s correlation coefficients were calculated using geometric features extracted from pairwise comparison metrics in the radiologists’ segmentations. Wilcoxon tests indicated that the CNN segmentation model satisfactorily matched the performance of the radiologists regarding sensitivity, specificity, the Dice coefficient, Cohen’s kappa, and 95% Symmetric Hausdorff Distance (*P* > 0.05). The Dice coefficient and Cohen’s kappa had the best values in Radiologist 2-CNN (0.83; 0.82); the specificity had the best values in Radiologist 3-CNN (0.99); and the 95% Symmetric Hausdorff Distance had the best values in Radiologist 1-CNN (1.19 mm) (**Table 3**). The box-plot diagrams show the intra- and interrater consistency of the radiologists and the CNN model compared to the radiologists (**Figure 6**). The Pearson’s correlation coefficients showed a strong relationship between the area, major and minor axis length between all of the observers (radiologists vs. CNN model *r* = 0.98, 0.97, 0.99). As shown in Bland–Altman plots, the differences between the CNN model and the three radiologists in segmentation area, major axis length, and minor axis length were almost 0 (**Figure 7**).

**Table 3:** Mean values of pairwise comparison metrics between all observers.

	Dice	Cohen’s kappa	95% HD	Specificity	Sensitivity
Radiologists 1-2	0.79	0.74	1.45 mm	0.98	**0.91**
Radiologists 2-3	0.81	0.79	1.34 mm	0.98	0.84
Radiologists 1-3	0.78	0.80	1.26 mm	0.95	0.76
Radiologists 1-CNN	0.80	0.81	**1.19mm**	0.96	0.84
Radiologists 2-CNN	**0.83**	**0.82**	1.29 mm	0.97	0.85
Radiologists 3-CNN	0.82	0.81	1.44 mm	**0.99**	0.77

The best values are shown in bold. The radiologists are numbered 1, 2, 3, and the model is labeled CNN.

### Diagnostic performance of the quantitative SWE parameters, US CNN model, and SWE CNN models for predicting breast cancer in the training and the validation cohorts

The diagnostic performances of quantitative SWE parameters, US CNN models, and SWE CNN models in both the training and the validation cohorts are summarized in **Table 4**.

**Table 4:** Performance summary of SWE CNN model, US+SWE CNN models and quantitative SWE parameters for predicted breast cancer in the training and validation cohort.

		SWE CNN models							US + SWE CNN models							US CNN models	SWV1	SWV5
		Internal	0.5 mm	1.0 mm	1.5 mm	2.0 mm		ROI	Internal	0.5 mm	1.0 mm	1.5 mm	2.0 mm	ROI				
Subgroup of lesions ≤ 15 mm (n= 216 )																		
AUC	T	0.65	0.70	0.81	0.76	0.75		0.74	0.81	0.89	**0.94**	0.92	0.90		0.87	0.75	0.70	0.79
	V	0.59	0.66	0.75	0.65	0.64		0.65	0.79	0.85	**0.91**	0.89	0.88		0.82	0.77	0.65	0.73
ACC%	T	64.23	72.46	79.26	74.70	73.56		70.71	75.65	82.97	**88.67**	87.32	85.45		84.57	75.54	78.64	81.89
	V	62.45	68.38	74.49	72.46	72.23		69.55	74.11	80.43	**85.54**	85.54	82.24		80.98	72.55	74.61	73.57
SEN%	T	47.65	63.69	68.86	66.85	65.64		64.53	57.53	65.64	**78.53**	76.58	70.32		69.63	77.33	65.52	65.63
	V	45.42	61.42	62.97	64.53	63.52		62.37	55.36	60.48	**77.22**	75.22	67.73		65.53	75.26	63.63	62.73
SPE%	T	68.23	73.63	82.52	75.64	74.63		72.52	74.66	79.64	**85.63**	84.93	85.50		83.57	76.58	67.53	79.33
	V	66.53	69.93	78.53	72.25	71.55		68.42	73.52	74.74	**82.52**	81.86	81.93		79.87	71.48	62.53	75.78
PPV%	T	67.75	73.97	77.64	75.90	74.94		72.73	76.33	82.53	**85.85**	84.63	77.63		75.44	76.34	71.64	76.53
	V	62.77	70.53	73.28	71.63	70.64		68.28	74.77	80.27	**82.12**	81.73	79.24		74.24	73.64	70.53	72.63
NPV%	T	65.75	67.85	75.66	74.63	72.64		70.64	72.74	75.72	**77.14**	76.84	74.35		73.63	74.86	71.63	74.25
	V	62.65	65.66	73.67	73.32	70.35		66.67	70.53	73.02	**73.54**	72.53	71.89		70.52	73.67	68.52	72.55
Subgroup of 15 mm > lesions ≤ 25 mm (n= 778 )																		
AUC	T	0.69	0.73	0.76	0.78	0.85		0.78	0.82	0.86	0.91	0.93	**0.96**		0.91	0.76	0.75	0.81
	V	0.64	0.68	0.70	0.73	0.81		0.70	0.75	0.81	0.85	0.87	**0.93**		0.86	0.73	0.71	0.76
ACC%	T	67.33	68.43	72.32	75.46	82.64		75.34	78.64	80.63	85.64	88.69	**91.34**		90.45	81.43	76.64	80.32
	V	64.43	64.76	69.76	72.42	78.87		73.53	75.75	76.75	83.63	85.85	**90.76**		88.65	77.64	75.74	78.34
SEN%	T	46.53	57.35	60.63	64.35	66.24		61.53	70.53	71.74	72.64	74.53	**75.63**		74.53	64.53	63.63	64.33
	V	44.43	55.53	57.63	60.42	63.44		56.63	65.53	67.53	66.53	70.53	**72.53**		70.53	58.42	59.34	63.53
SPE%	T	71.74	73.64	76.53	75.68	80.33		74.78	75.85	78.96	84.36	84.74	**86.98**		85.64	76.74	66.84	74.26
	V	66.47	70.63	72.35	72.87	76.85		72.25	73.64	72.63	80.75	81.85	**84.22**		83.63	73.45	62.67	70.75
PPV%	T	70.53	71.53	75.63	76.76		80.56	73.64	83.65	83.76	85.64	85.98	**87.96**		84.53	78.64	75.76	79.54
	V	65.75	68.82	72.73	73.81	76.73		70.97	78.14	80.14	83.34	81.34	**85.34**		82.53	74.22	73.25	74.82
NPV%	T	64.54	66.76	67.54	65.53	76.87		65.78	74.65	76.64	77.75	79.94	**81.26**		80.09	76.75	71.64	75.84
	V	61.85	63.64	62.75	64.74	74.93		62.97	70.93	72.64	73.36	75.13	**77.57**		76.32	73.21	68.96	73.26
Subgroup of lesions > 25mm (n=274 )																		
AUC	T	0.66	0.74	0.78	0.80	0.84		0.81	0.84	0.88	0.91	0.94	**0.95**		0.92	0.77	0.72	0.82
	V	0.63	0.67	0.70	0.74	0.78		0.70	0.75	0.78	0.87	0.89	**0.91**		0.87	0.75	0.68	0.74
ACC%	T	65.45	67.63	71.77	73.53	80.34		71.76	79.75	80.74	83.64	85.64	**86.65**		86.32	78.79	77.90	78.06
	V	62.25	64.75	69.85	70.74	77.73		69.64	75.63	78.74	80.25	83.22	**84.23**		83.63	75.52	76.73	74.86
SEN%	T	50.64	53.65	65.83	68.85	69.34		67.53	56.33	67.73	76.53	78.58	**80.32**		75.63	76.53	74.59	79.61
	V	48.52	50.66	63.27	66.83	65.73		64.33	54.78	65.37	73.72	75.72	**77.38**		72.63	74.27	71.23	76.36
SPE%	T	72.64	75.64	73.78	76.86	82.63		75.53	74.53	78.57	82.64	84.35	**87.44**		84.53	76.63	74.53	80.53
	V	68.87	72.54	70.98	72.29	77.64		70.28	73.74	73.88	80.75	83.98	**84.75**		80.37	73.96	73.64	74.23
PPV%	T	67.85	70.65	73.66	75.63	82.43		72.64	82.64	83.64	84.23	86.04	**86.57**		84.35	80.42	76.53	79.64
	V	64.65	67.37	70.75	72.64	77.74		70.83	78.44	80.85	82.75	83.44	**84.37**		83.25	76.74	73.67	75.36
NPV%	T	65.64	67.54	70.63	73.53	83.65		72.64	79.64	80.53	81.04	82.03	**87.64**		80.74	76.37	73.53	79.84
	V	62.53	62.79	66.95	70.95	76.96		69.85	73.26	74.86	75.74	75.98	**86.43**		73.44	74.75	70.64	75.89

The best values are shown in bold. T, training cohort; V, validation cohort; ROI, whole SWE ROI box image; SWV, shear wave velocities.

Among these three single models, the 1.0 mm SWE image CNN model had the highest area under curve (AUC), accuracy (ACC), sensitivity, and specificity for predicting breast cancer in the subgroup with MD ≤15 mm both in the training and in the validation cohort (0.81, 79.26%, 68.86%, 82.52% *vs.* 0.75, 74.49%, 62.97%, 78.53%).

In the subgroups with 15 mm <MD ≤25 mm and MD >25 mm, the 2.0 mm SWE image CNN model had the highest AUC, ACC, sensitivity, and specificity both in the training cohort (15 mm <MD ≤25 mm: 0.85, 82.64%, 66.24%, 80.33%; MD >25 mm: 0.84, 80.34%, 69.34%, 82.63%) and the validation cohort (15 mm <MD ≤25 mm: 0.81, 78.87%, 63.44%, 76.85%; MD >25 mm: 0.78, 77.73%, 65.73%, 77.64%).

**Figure 8:**
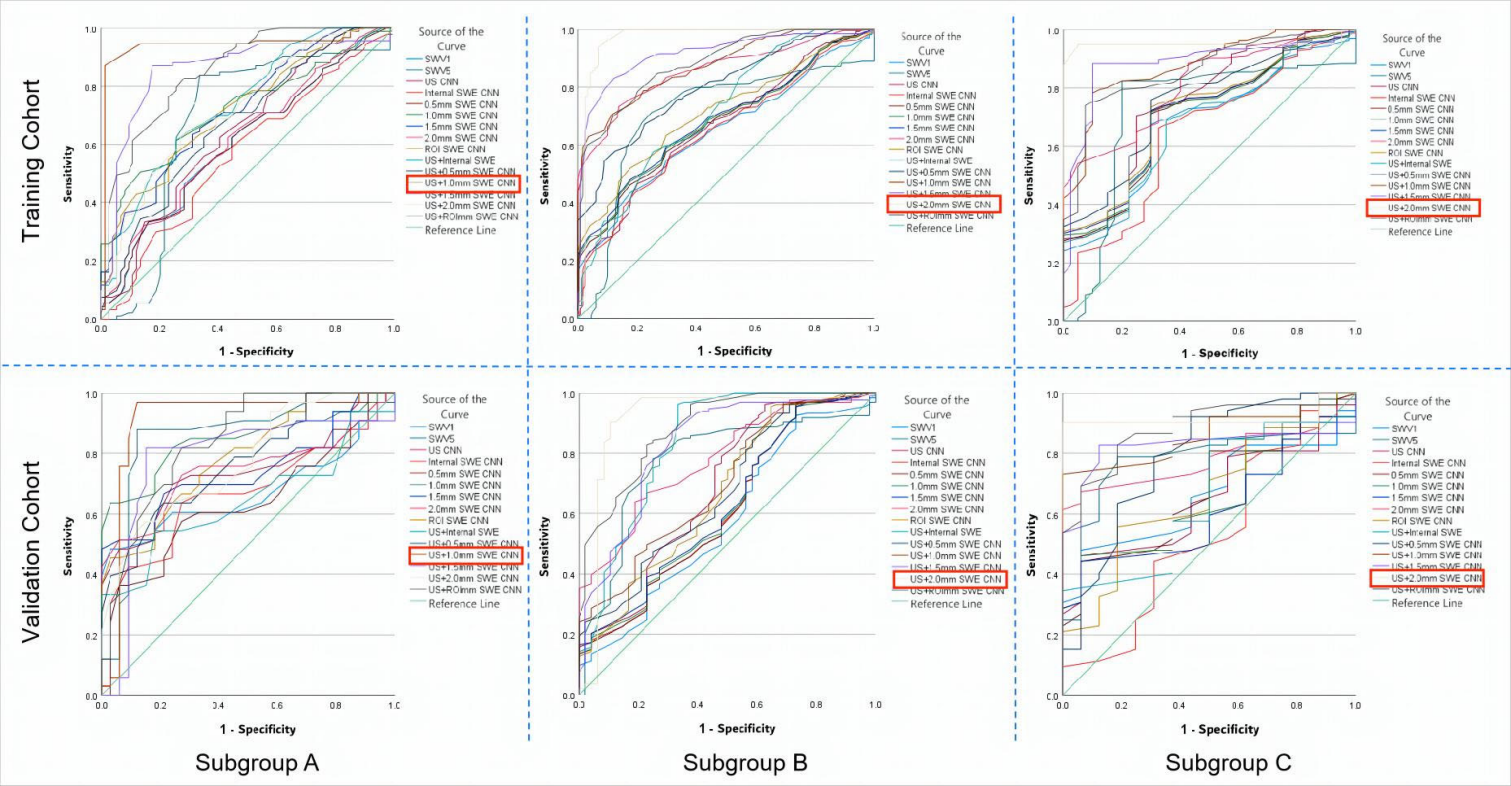
The ROC curves of the prediction CNN models and the quantitative SWE parameters (SWV1 and SWV5) in both the training and the validation cohorts for the three subgroups. **(A)** Based on the ROC curves of the subgroup with MD ≤15 mm, the US + 1.0 mm SWE CNN showed the highest AUCs in the training (0.94) and in the validation (0.91) cohorts. **(B)** From the ROC curves of the subgroup with 15 mm< MD≤ 25 mm, the US + 2.0 mm SWE CNN showed the highest AUCs in the training (0.96) and in the validation (0.93) cohorts. **(C)** From the ROC curves of the subgroup with MD >25 mm, the US + 2.0 mm SWE CNN showed the highest AUC in both the training (0.95) and the validation (0.91) cohorts. ROC, receiver operating characteristic; AUC, area under the ROC curve; MD, maximum diameter of the lesion. The best values are shown in red box mark.

Regardless of the grouping method, the AUCs and ACC of the SWE image CNN models were all better than SWV5 and the US CNN model in predicting breast cancer (all *P* < 0.05), except for the 0.5 mm and the internal SWE image CNN models. There was no significant difference between the SWV5 and the US CNN model in sensitivity, specificity, and AUCs (all *P* > 0.05, **Figure 8**).

### Diagnostic performance of the US + SWE dual-modal CNN model for predicting breast cancer in the training and the validation cohorts

In both the training and the validation cohorts, the overall performances of the US + SWE image CNN models were slightly higher than those of the corresponding single-image CNN models.

The US CNN + 1.0 mm SWE model achieved the highest AUC for MD ≤15 mm both in the training cohort (0.94) and in the validation cohort (0.91) (**Figure 8**). In the subgroup with MD ≤15 mm, the US + 1.0 mm SWE CNN model achieved the highest ACC, sensitivity, and specificity both in the training cohort (88.67%, 78.53%, 85.63%, respectively) and in the validation cohort (85.54%, 77.72%, 82.52%, respectively).

In the subgroups with 15 mm <MD ≤25 mm and MD >25 mm, the US CNN + 2.0 mm SWE model achieved the highest AUCs both in the training cohort (0.96, 0.95, respectively) and in the validation cohort (0.93, 0.91, respectively) (**Figure 8**). Comparing the results obtained by the US + SWE dual-modal CNN model to those of the US images CNN model, there were 6.2%, 5.7%, and 8.7% average percentage increases for ACC, sensitivity, and specificity, respectively, where the improvement in specificity was significant, indicating that the dual-modal CNN model can improve specificity without loss of sensitivity for classifying breast cancer.

Similarly, the US + 2.0 mm SWE CNN model achieved the highest ACC, sensitivity, and specificity in both the training and the validation cohorts for 15 mm <MD ≤25 mm (91.34%, 75.63%, 86.98% and 90.76%, 72.53%, 84.22%) and MD >25 mm (86.65%, 80.32%, 87.44% and 84.23%, 77.38%, 84.75%).

## Discussion

The most important contribution of this work is the introduction of the dual-modal CNN architecture to predict breast cancer. We adopted the deep learning algorithm-assisted strategy for clinical diagnosis of breast cancer based on the automatic segmentation of peritumoral region on ultrasound SWE images. Our dual-modal CNN architecture improved the SWE diagnostic accuracy of breast cancer, reaching 90.76%.

The ACR-BIRADS category is based on the morphological features visible on US images. This approach has high sensitivity but relatively low specificity, thereby leading to unnecessary biopsy and excessive diagnosis (19, 20). The SWE examination has been reported to be able to increase specificity and sensitivity for predicting breast cancer (8–10). Our results showed that SWV5 values were higher than SWV1 values in the malignant group (all *P* < 0.001), while there was no significant difference between SWV1 and SWV5 in the benign group. SWV5 had higher AUCs than SWV1 both in the training and in the validation cohorts for predicting breast cancer, regardless of the subgroup. This demonstrated that stiffness that includes peritumoral tissue is a better indicator of breast cancer, which is consistent with previous studies (21–24), because the peritumoral region of breast cancer has abnormally stiff collagen fibers, which is related to cancer fibroblasts, as well as infiltration of cancer cells into the surrounding tissue (25, 26). However, based on the traditional SWE technology, it is difficult to accurately select the surrounding tissues and obtain accurate peritumoral stiffness value. This may be the reason why SWE technology has not yet been widely applied for clinical diagnosis of breast cancer (27, 28). Few studies have evaluated the value of intra- and peritumoral regions in the prediction of breast cancer, and no studies have shown the diagnostic efficacy of CNNs segmenting different widths of peritumoral region of breast cancer.

In the subgroup with MD ≤15 mm, comparing the results obtained by dual-modal CNN models integrating US and SWE images with those of just using US or SWE images single CNN models, the accuracy, sensitivity, and AUC were all improved, with specificity increasing most significantly (average 7.8%). Such an improvement in specificity may be because SWE images provide information on the stiffness of lesions, complementing the US image diagnosis of breast cancer from another dimension. The US + 1.0 mm SWE model achieved the highest AUC in diagnosing breast cancer both in the training (0.94) and in the validation cohorts (0.90).

In the subgroups with 15 mm <MD ≤25 mm and MD >25 mm, the dual-modal US + SWE model still had higher AUC, accuracy, sensitivity, and specificity than any of the single-parameter CNN models. The US + 2.0 mm SWE model achieved the highest AUC in predicting breast cancer both in the training (0.96; 0.95) and in the validation cohort (0.93; 0.91). These results show that the most effective SWE image region for predicting breast cancer is the area containing 2.0 mm of peritumoral tissue. Similarly, for the lesions with the maximum diameter ≤15 mm, the most effective image region for SWE to predict breast cancer is the area containing 1.0 mm of peritumoral tissue. Regardless of the group, the 0.5 mm SWE CNN model and the US + 0.5 SWE dual-modal CNN model showed no significant difference from the corresponding lesion’s internal SWE CNN model, possibly because stiffness of the 0.5 mm peritumoral tissue was very close to intratumoral tissue stiffness.

The reliability and repeatability of automatic segmentation are crucial. In our research, the segmentation CNN model achieved stable consistency with all three radiologists in terms of Dice coefficient and Cohen’s kappa. The difference of each observer in the segmentation area and the maximum diameter of the transverse and longitudinal sections of the lesions was very close to 0, indicating that the CNN segmentation model has excellent consistency with radiologists.

The CNN architecture does not require manual input of radiomics signatures, but automatically selects useful features from US images for classification; this improves diagnostic performance and efficiency while minimizing artificial false-positive or false-negative errors (29–32). However, it is unknown which region of the CNN model based on US and SWE images can improve the diagnostic efficiency of breast cancer, so it is called a black-box learning method, which may lead to ambiguous interpretation of CNN features (33, 34). In our study, we constructed an image segmentation model to automatically segment the effective area (the optimal peritumoral width) on SWE images that is conducive to the diagnosis of breast cancer, and then integrated the automatic segmentation model into our CNN prediction model, thereby breaking the previous CNN black-box learning mode and greatly improving the efficiency of the CNN model in the diagnosis of breast cancer.

Our results showed that the CNN models combining US images with SWE images containing 1 mm or 2 mm of the peritumoral tissue were superior to whole SWE ROI image models in predicting breast cancer, indicating that the effective SWE region of breast cancer was the area covering a certain width of peritumoral tissue. Moreover, the lesions with different diameters have differential infiltration into the peritumoral tissue. Our CNN model based on automatic segmentation of SWE images of peritumoral tissue can automatically identify effective regions of breast lesions’ SWE image, thereby improving the work efficiency of radiologists and reducing their SWE measurement workload.

There are some limitations to this study. First, this study only considered ACR-BIRADS 4 lesions, there are some deviations in the study samples. Second, all images and data came from a single center. Therefore, a larger data cohort acquired from multiple centers with different models of US equipment is necessary to create a more comprehensive training cohort.

## Conclusion

In conclusion, the dual-modal CNN models based on the combination of US images and peritumoral region’s SWE allow for accurate prediction of breast cancer. Moreover, when lesion’s diameter is ≤15 mm, the best diagnostic SWE image area for predicting breast cancer contains 1.0 mm of the peritumoral region. When lesion diameter is >25 mm, or between 15 mm and 25 mm, the SWE image containing 2.0 mm of the peritumoral region is the optimal diagnostic area for predicting breast cancer.

## Data availability statement

The raw data supporting the conclusions of this article will be made available by the authors, without undue reservation.

## Ethics statement

The studies involving human participants were reviewed and approved by USTC. Written informed consent for participation was not required for this study in accordance with the national legislation and the institutional requirements.

## Author contributions

LX, ZL, CP, XL, Y-YC and LH participated in literature search, data acquisition, data analysis, or data interpretation. LX and LH conceived and designed the study, and critically revised the manuscript, performed the research, wrote the first draft, collected and analyzed the data. LX, N-AH, and LH participated in paper writing and revised the manuscript. All authors contributed to the article and approved the submitted version.
